# Sensitivity and specificity of four screening sleep-disordered breathing tests in patients with and without cardiovascular disease

**DOI:** 10.5935/1984-0063.20200104

**Published:** 2021

**Authors:** Sandra Brigitte Amado-Garzón, Alvaro J Ruiz, Martín Alonso Rondón-Sepúlveda, Patricia Hidalgo-Martínez

**Affiliations:** 1 Department of Internal Medicine, Hospital Universitario San Ignacio Bogota, Colombia.; 2 Department of Internal Medicine, Pontificia Universidad Javeriana Medical School Bogota, Colombia.; 3 Department of Clinical Epidemiology and Biostatistics Pontificia Universidad Javeriana Medical School Bogota, Colombia.; 4 Pulmonology Unit - Department of Internal Medicine Hospital Universitario San Ignacio Bogota, Colombia.

**Keywords:** Sleep Apnea, Respiratory Disorders, Screening, Scales, Questionnaire, Altitude

## Abstract

**Objectives:**

Polysomnogram is the gold standard for the diagnosis of sleep-disordered breathing (SDB); a sensitive and specific alternative strategy would be ideal, due to its low availability, and screening patients at high risk of OSA is very important. This study aimed to determine the operating characteristics of screening tests in patients with and without cardiovascular disease (CVD).

**Material and Methods:**

Epworth sleepiness scale (ESS), Berlin, STOP-bang and Pittsburgh sleep quality index (PSQI) were applied in adults with and without cardiovascular disease in three Colombian cities, as well as anthropometric measurements and a polysomnogram. Operating characteristics were calculated for each test and the best cut-off values in patients with and without CVD were obtained.

**Results:**

964 patients (median age: 58), 662 with and 302 without CVD were included. The prevalence for SDB (AHI =5) were 43.4 % (OSA), 16.2% (central apnea), and 12.4 % (other). In patients without CVD, the highest sensitivity for OSA and central apnea was for PSQI (80-85%). The highest specificity was for STOP-bang (68%) and Berlin (78.6%). In CVD the best sensitivity was for PSQI (81.9%) followed by Berlin (71.9%) and the best specificity for STOP-bang (82.1%). No isolated questionnaire showed good diagnostic performance (AUC=0.6) and the cut-off values had no variations except for ESS.

**Conclusion:**

Screening tests showed low operating characteristics for the diagnosis to SDB, but better performance in patients with CVD. They are not recommended as the only diagnostic test, but they can be useful to guide the initial diagnostic process.

## INTRODUCTION

The frequency of sleep disorders in the world is high; it have been reported as 56% in the US population, 31% in Western Europe, and 23% in Japan^1^ and Latin America is not the exception: studies in different countries have estimated the prevalence of sleep disorders and specifically for obstructive sleep apnea (OSA) with values around 19% in Colombia^2^ and 30% of the studied population in Sao Paulo, Brazil^3^; in the PLATINO study the estimated prevalence of OSA were in Santiago of Chile 8.8% and 5.5%; Mexico City 4.4% and 2.4%; Montevideo 3.7% and 0.5%; and Caracas 1.5% and 2.4% for men and women, respectively^4^.

OSA and central sleep apnea (CSA) are clearly associate with cardiovascular disease (CVD) and patients have increased risk of developing CVD and having worse outcomes; OSA is associated with an increased incidence of high blood pressure (HBP), type 2 diabetes mellitus, atrial fibrillation, heart failure, coronary heart disease, stroke, and death^5^ and specifically in patients with severe OSA, an increase in fatal and non-fatal cardiovascular events has been reported (adjusted HR of 2.8 and 3.1, respectively)^6, 7^. On the other hand, the prevalence of OSA and CSA is higher in population with CVD^8^; it is ideal to have access to accessible and highly sensitive tests in these patients.

The polysomnogram (PSG type 1) is the gold standard for the diagnosis of sleep-disordered breathing (SDB) and is defined as an apnea-hypopnea index (AHI) greater than 5 per hour with symptoms or greater than 15 without symptoms. The American Academy of Sleep Medicine (AASM) recommends a polysomnogram study in high-risk patients with coronary heart disease, cerebrovascular disease, arrhythmia or heart failure who have symptoms suggesting a sleep disorder may be present^9, 10^.

The availability of sleep laboratories, as well as clinical diagnostic suspicion is low. As such, sleep disorders are frequently underdiagnosed. Likewise, there are logistical difficulties for the exam such as the waiting time for the appointment, transportation, and the time it takes to perform the exam.

Although the studies show a poor diagnostic performance of the screening questionnaires, they are frequently used in Colombia due to the limited availability of the gold test and there are some characteristics such as altitude that suggest that their performance may be different. While clinical signs or questionnaires offer the advantage of being convenient, quick, and inexpensive, the discriminatory power for the diagnosis of SDB by themselves has been shown to be low. However, most of the published studies on this topic come from sleep laboratories in populations that had a high prevalence of OSA and are therefore more likely to be symptomatic. That could change the performance of the test in this population; this could be the case in CVD patients. Some studies have shown that, for example, there are gender differences in the reporting of classic OSA symptoms and in the performance of sleep questionnaires in adults^11, 12, 13^. In Colombia, there is a high frequency of sleep disorders^2^ and CVD is considered the main cause of mortality^14^; but their relationship is not known.

There are few studies that include the general population; the objective of this study is to describe the operational characteristics of four screening tests to diagnose sleep disorders, compared to the polysomnogram as the gold standard in patients with and without CVD.

## MATERIAL AND METHODS

### Study design

This is a study of operative characteristics of four sleep disorder screening scales; a retrospective analysis was carried out. It included 964 adults over the age of 18; 662 with associated CVD attending the Heart Institute, from three cities in Colombia (Bogotá (2,630 m.a.s.l), Santa Marta (2 m.a.s.l) and Bucaramanga, (959 m.a.s.l)), and the rest without CVD. Patients with mental illnesses that limited the filling of questionnaires were excluded. The protocol was approved by the research ethics committee of the Pontificia Universidad Javeriana in Bogota.

### Measuring instruments

Each participant answered a 40-item questionnaire before the polysomnogram was carried out. This questionnaire included demographic data, contact information and the screening tests: Epworth sleepiness scale (ESS), Pittsburgh sleep quality index (PSQI), Berlin and STOP-bang questionnaire.

Overnight PSG were performed using the standard PSG, (an Alice 5 equipment; Philips Respironics, 1010 E.E.U.U) was used; electroencephalogram, electrooculograms, chin electromyogram, nasal pressure detected by airflow pressure transducer, respiratory effort, electrocardiography, pulse oximetry and position were recorded. All the sleep scoring and respiratory events were analyzed using software and analyzed manually by a sleep specialist (who did not know the results of the questionnaires applied to the participants), in accordance with the standards established by the American Academy of Sleep Medicine (AASM)^15^.

CVD was defined as the presence of structural heart disease, or heart disease diagnosed in follow-up medical visits, including: heart failure of ischemic or valvular origin, coronary heart disease, and arrhythmia; supported with studies such as electrocardiogram, echocardiogram, myocardial perfusion images, Holter electrocardiogram or cardiac catheterization. HBP was considered if a patient was previously diagnosed with hypertension or if he was treated with antihypertensive drugs and was considered as a different group. Other medical conditions including diabetes, hyperlipidemia, chronic obstructive pulmonary disease (COPD), thyroid disease, neurologic disease, and psychological drug administering history were also recorded.

Considering that an additional measures was taken for the ESS in the sleep laboratory before the polysomnogram, the correlation and concordance index between the two measures was calculated.

For the ESS a score =11 was considered as abnormal^16^; for the Berlin questionnaire, high probability for sleep apnea was defined as a score =2 out of the 3 categories^17^; for the PSQI, a cut off of 5 was used to categorize “good sleepers” (<5) and “bad sleepers” (>5)^18^; these scales were validated in Colombia. The STOP-bang questionnaire for sleep apnea was developed to assess the likelihood of OSA in the surgical field and has been validated in the general population; a score =3 indicated intermediate or high risk for OSA^19^.

Regarding the polysomnogram-based definition, a diagnosis of OSA is considered when there is an AHI =5 per hour with a majority of events obstructive with associated symptoms or greater than 15 without symptoms. For the diagnosis of CSA an AHI =5 per hour and it is required that more than 50% of the events be classified as of central origin. According to the AHI, the severity of the disease is classified as follows: mild (AHI>5 and <15); moderate (>15 and <30) and severe (>30/hour)^20^.

### Statistical analysis

All data were analyzed using STATA (14.0) (StataCorp; College Station, TX, USA), normality of variables was tested by the Shapiro-Wilk W test. An unpaired, two-tail t-test and a chi-squared or Fisher’s exact test analysis were used for comparison between the groups. Operating characteristics (sensitivity (S), specificity (E) and predictive values) for the four questionnaires were calculated according to the severity of each of the disorders, data obtained from the PSG report and the proposed cut-off values for each of the questionnaires. ROC (receiver operating characteristic) curves were constructed for the diagnosis of SDB in patients with CVD and the curves were compared using the equality test of 2 or more ROC areas, confidence intervals were estimated at 95%. Likewise, it was sought if there were better S and E cut-off points for the diagnosis of OSA and CSA for each of the tests in the group of patients with CVD, by means of non-parametric analysis (De Long) and the Liu’s method^21^.

## RESULTS

### General characteristics

Table 1 shows the demographic and clinical characteristics of the patients. 964 patients from urban areas were included, 66% were from the city of Bogotá, 25% from Bucaramanga and 9% from Santa Marta. 662 adults had CVD (arrhythmia, heart disease of any type or both) and 302 came from the general population. Unlike patients without CVD, the majority of patients with CVD were men (65.2%) with a median age of 63 years. The distribution of body mass index (BMI) was similar in both groups, the majority in the overweight range. The frequency of BMI over 30 was 10.7% for men and 9.7% for women. Neck and abdominal perimeter median values were greater in the patients with CVD and was 98 cm (DS11.9) in the general population.

**Table 1:** Sample description according to the presence of cardiovascular disease.

	With CVD n (631)	Without CVD n (326)	p-value
**Age, median years (IQR)**	63 (55-71)	47(34-57)	<0.001
**Male sex, n (%)**	416(65.2)	140(42.9)	<0.001
**BMI, kg/m2, n (%)**			
<18	1 (0.1)	2(0.6)	0.078
18-24.9	216(33.9)	123(37.7)	
25-29.9	297(46.6)	127(39.0)	
>30	124(19.4)	74(22.7)	
**Neck perimeter, cm, n (%)**	346(54.3)	120(36.8)	<0.001
=>40 cm			
**Abdominal perimeter, cm, n (%) >90 cm**	525(82.3)	234(71.8)	<0.001
*OSA, n (%)			
Mild	135(49.3)	77(53.5)	0.597
Moderate	83(30.3)	37(25.7)	
Severe	56(20.4)	30(20.8)	
*CSA, n (%)			
Mild	38(31.1)	9(26.5)	0.869
Moderate	33(27.1)	10(29.4)	
Severe	51(41,8)	15(44.1)	
**Epworth**	293(45.9)	187(57.3)	0.001
=>11			
**Berlin**	442(69.3)	99(30.4)	<0.001
=>2 categories			
**Pittsburgh**			
<5: Normal	135(21.1)	65 (20.0)	0.721
5-7: Medical attention	206(32.3)	112(34.3)	
8-14: Attention and treatment	246(38.6)	118(36.2)	
14-21: Severe sleep disorder	51(8.0)	31(9.5)	
**STOP-bang**			
=>3 High risk	540(84.6)	176(54)	<0.001
**Comorbidities, n (%)**			
HBP	495(51.3)	53(5.5)	<0.001
Diabetes	140(14.5)	12(1.24)	<0.001
Depression	111(11.5%)	25(2.6)	<0.001
Anxiety	103(10,7)	33(3.4)	0.011
Hypothyroidism	110(11.4)	39(4.05)	0.032
COPD	40(4.15)	2(0.21)	<0.001
GERD	68(7.05)	41(4.25)	0.374

Notes: IQR = Interquartile range; BMI = Body mass index; OSA = Obstructive sleep apnea; CSA = Central sleep apnea; HBP= High blood pressure; COPD = Chronic obstructive pulmonary disease; GERD = Gastro esophageal reflux disease; CVD = Cardiovascular disease; *Results by PSG.

### Comorbidities

Regarding comorbidities, 49% had heart disease of any etiology; 3% had some type of arrhythmia (either brady or tachyarrhythmia). 423 patients had coronary heart disease, the majority were men (68%) and 76% had some SDB (41% with OSA the mild majority and 19% with predominantly severe CSA).

The prevalence of HBP in the total group was 56.8%; other comorbidities were diabetes (16%), hypothyroidism (15%), depression and anxiety (14%), and chronic obstructive pulmonary disease (COPD) (4.3%), all of them more frequent in the CVD group (statistically significant) except for gastro esophageal reflux disease (GERD) (11.3%, p=0.374). Table 1 shows the difference in frequencies according to the presence of CVD.

### SDB

The prevalence of SDB defined as AHI=5 was 72% in the general population. Most patients presented AHI between 5 and 15 per hour, that is, mild, with a similar distribution in patients with and without CVD (Table 1). As for each disorder, the frequency OSA and CSA was 43.4% and 16.2%, respectively.

The percentage of cases of OSA (24.9% vs. 8.6%; *p*>0.01) and CSA (56.7% vs. 22.7%; *p*<0.01) classified as severe was higher in Bogotá than other cities. The Cheyne Stokes pattern was present in 54 patients (5.6% of the sample); 15 of them had OSA and 39 CSA.

Of the patients with HBP, 45% had SDB; 15% of them with an AHI greater than 30; the frequency of hypothyroidism and diabetes was also higher in these patients.

### Operating characteristics of the tests

For the ESS, the correlation and concordance index between the two performed measures (surveys and sleep laboratory) was 0.74 (95% CI: 0.71-0.76). Table 2 shows the operating characteristics of the different questionnaires for the diagnosis of SDB. For the patients without CVD, it was found that for the diagnosis of OSA and CSA, the highest sensitivity (S) was found the PSQI, with values between 80 and 85%. The highest specificity (E) for the diagnosis of OSA was for the Berlin questionnaire (78.6%).

**Table 2:** Operating characteristics for sleep-disordered breathing according to the presence of cardiovascular disease.

	OSA		CSA	
CI 95 %	Without CVD	With CVD	Without CVD	With CVD
**Epworth**				
Prevalence %	44 (39-49.7)	43 (39-46.9)	10 (7.3-14.3)	19(16-22.4)
Sensitivity %	58.3(49.8-66.5)	47.4(41.4-53.5)	47.1 (29.8-64.9)	47.5(38.4-56.8)
Specificity %	43.4(36.1-50.9)	55.2(49.9-60.4)	41.4 (35.7-47.3)	54.5 (50-58.8)
PPV %	44.9(37.7-52.3)	44.4(38.6-50.3)	8.56(4.97-13.5)	19.8 (14.4-24.8)
NPV %	56.8 (48.2-65.2)	58.3(52.9-63.5)	87.1 (80.3-92.1)	81.4(76.9-85.4)
ROC area	0.50(0.45-0.56)	0.51(0.47-0.55)	0.44 (0.35-0.53)	0.51 (0.46-0.55)
**Berlin**				
Prevalence %	44 (39-49.7)	43(39-46.9)	10(7.3-14.3)	19(16-22.4)
Sensitivity %	41.7(33.5-50.2)	71.9(66.2-77.1)	41.2(24.6-59.3)	69.7 (60.7-77.7)
Specificity %	78.6(71.9-84.3	32.7(27.9-37.8)	70.9(65.3-76)	30.8 (26.9-35)
PPV %	60.6(50.3-70.3)	44.6(39.9-49.3)	14.1(7.95-22.6)	19.2(15.7-23.2)
NPV %	63(56.4-69.3)	60.7 (53.5-67.6)	91.2(86.7-94.5)	81.1(74.9-86.3)
ROC area %	0.60 (0.55-0.65)	0.52(0.48-0.55)	0.56(0.47-0.64)	0.50 (0.45-0.54)
**STOP-bang**				
Prevalence %	44(39-49.7)	43 (39-46.9)	10(7.3-14.3)	19 (16-22.4)
Sensitivity %	29.2(21.9-37.3)	12(8.44-16.5)	32.4 (17.4-50.5)	16.4(10.3-24.2)
Specificity %	40.7(33.5- 48.2)	82.1(77.8- 85.9)	52.4 (46.5- 58.2)	84.9(81.5-87.9)
PPV %	28(21-35.9)	33.7(24.4-43.9)	7.33(3.72-12.7)	20.4 (12.9-29.7)
NPV %	42(34.7-49.7)	55.4(51.1-59.6)	86.9(81-91.5)	81.1 (77.6-84.3)
ROC area	0.34 (0.29-0.40)	0.47(0.44-0.49)	0.42 (0.33-0.50)	0.50(0.45-0.54)
**Pittsburgh**				
Prevalence %	43 (39-46.9)	44(39-49.7)	19(16-22.4)	10 (7.3-14.3)
Sensitivity %	80.3 (75.1-84.8)	81.9(74.7-87.9)	81.1(73.1-87.7)	85.3(68.9-95)
Specificity %	22.3(18.1-26.9)	21.4(15.7-28.1)	21.7 (18.2-25.5)	20.5 (16.1-25.6)
PPV %	43.7 (39.4-48.2)	45.2(39.1-51.5)	19.7 (16.3-23.4)	11.1 (7.57-15.6)
NPV %	60 (51.2-68.3)	60(47.1-72)	83(75.5-88.9)	92.3 (83-97.5)
ROC area %	0.51 (0.48-0.54)	0.51(0.47-0.56)	0.51(0.47-0.55)	0.52 (0.46-0.59)

**Notes:** OSA = Obstructive sleep apnea; CSA = Central sleep apnea; CVD = Cardiovascular disease; CI = Confidence interval; PPV = Positive predictive value; NPV = Negative predictive value; ROC = Receiver operating characteristic.

In patients with CVD, the best S for the diagnosis of OSA and CSA was for the PSQI (81.9%) followed by the Berlin questionnaire (71.9%) and the best E for the STOP-bang questionnaire (82.1%). Specifically, in patients with coronary heart disease, E was better too for the STOP-bang questionnaire (86%) (Supplementary Table 1).

When calculating the operating characteristics for the SDB according to the AHI (Table 3), both for moderate to severe OSA and CSA, the highest S was maintained for the PSQI (79.2%), followed by Berlin. For diagnosis of moderate to severe OSA, STOP-bang showed E 90.8%, followed by ESS (50%).

**Table 3:** Operating characteristics for OSA and CSA according to severity (AHI)*.

OSA						
CI 95%	Prevalence %	Sensitivity %	Specificity %	PPV %	PNV %	ROC Area
**Epworth**						
Mild*	21 (19-24)	52.9 (45.9-59.9)	51.1 (47.4-54.7)	22.7 (19- 26.7)	80(76.1-83.4	0.52(0.48-0.55)
Moderate*	51 (46-55.6)	49.5(42.6-56.5)	47.1(40.1-54.1)	49.1 (42.2-56)	47.5(40.5-54.6)	0.48 (0.43-0.53)
Severe*	8.9 (7.2-10.9)	53.5 (42.4-64.3)	50.6(47.2-53.9)	9.58(7.1-12.6)	91.7(88.9- 94)	0.52 (0.46- 0.57)
**Berlin**						
Mild	21 (19-24)	68.9 (62.1-75.2)	47.4 (43.8-51)	26.2(22.6-30.2)	84.9 (81.1-8.1)	0.58(0.54-0.61)
Moderate	51 (46-55.6)	54.2(47.3-61.1)	31.1 (24.8-37.9)	44.7(38.6-51.1)	39.8 (32.1-47.8)	0.42 (0.38-0.47)
Severe	8.9(7.2-10.9)	73.3 (62.6-82.2)	45.6 (42.2-48.9)	8.07 (5.72-11)	94.6 (92-96.5)	0.59 (0.54-0.64)
**STOP-bang**						
Mild	21(19-24)	9.22 (5.64-14)	69.8 (66.4-73)	7.66 (4.68-11.7)	73.9 (70.5-77.1)	0.39(0.36-0.42)
Moderate	51 (46-55.6)	26.4 (20.6-32.9)	90.8 (86-94.4)	74.7 (63.3-84)	54.5 (49.1-59.9)	0.58(0.55-0.62)
Severe	8.9 (7.2-10.9)	6.98 (2.6-14.6)	72.4 (69.4-75.4)	2.42(0.89-5.19)	88.8 (86.3-91)	0.39 (0.36-0.42)
**Pittsburgh**						
Mild	21 (19-24)	82.5 (76.6-87.4)	21.6(18.8-24.7)	22.3(19.3-25.4)	82(76-87.1)	0.52 (0.49-0.55)
Moderate	51 (46-55.6)	79.2 (73.2-84.5)	17.5 (12.6-23.4)	49.7(44.2-55.2)	45 (33.8-56.6)	0.48(0.44-0.52)
Severe	8.9(7.2-10.9)	79.1(69-87.1)	20.7 (18.1-23.6)	8.9(6.98-11.1)	91 (86.1-94.6)	0.49(0.45-0.54)
**CSA**						
**Epworth**						
Mild	11(9.4-13.5)	48.6 (38.9-58.4)	50.1 (46.7-53.5)	11(8.38-14.2)	88.4(85.2-91.1)	0.49(0.44-0.54)
Moderate	30 (23-38)	44.7(30.2-59.9)	51.4 (41.6-61.1)	28.4 (18.5-40.1)	68.3 (57.1-78.1)	0.48 (0.39-0.56)
Severe	0.5 (0.44-0.57)	51.5(38.9-64)	50.3 (47-53.7)	7.08 (4.95-9-76)	93.4 (90.8-95.4)	0.50 (0.44-0.57)
**Berlin**						
Mild	11(9.4-13.5)	62.4 (52.6-71.5)	44.7 (41.3-48.1)	12.6 (9.89-15.7)	90.3 (87-93)	0.53 (0.48-0.58)
Moderate	30(23-38)	66 (50.7-79.1)	37.6(28.5-47.4)	31.3 (22.4-41.4)	71.9 (58.5-83)	0.51 (0.43-0.6
Severe	0.5 (0.44-0.57)	62.1 (49.3-73.8)	44.3 (41-47.6)	7.58 (5.49-10.1)	94.1 (91.4-96.1)	0.53 (0.47-0.59)
**STOP-bang**						
Mild	11 (9.4-13.5)	15.6(9.36-23.8)	73 (69.9-75.9)	6.85 (4-10.7)	87.2 (84.5-89.5)	0.44(0.40-0.48)
Moderate	30 (23-38)	29.8 (17.3-44.9)	84.4 (76.2-90.6)	45.2(27.3-64)	73.6 (65-81.1)	0.57 (0.49-0.64)
Severe	0.50 (0.44-0.57)	18.2(9.76-29.6)	73.7 (70.7-76.6)	4.84 (2.52-8.3)	92.5 (90.3-94.3)	0.46 (0.41-0.50)
**Pittsburgh**						
Mild	11 (9.4-13.5)	82.6 (74.1-89.2)	21.2 (18.5-24.1)	11.8(9.58-14.3)	90.5(85.6-94.2)	0.51 (0.48-0.55)
Moderate	30 (23-38)	80.9 (66.7-90.9)	17.4 (10.8-25.9)	29.7 (21.9-38.4)	67.9(47.6-84.1)	0.49 (0.42-0.55
Severe	0.50 (0.44-0.57)	81.8 (70.4-90.2)	20.9 (18.3-23.7)	7.07(5.3-9.1)	94 (89.8-96.9)	0.51 (0.46-0.56)

Notes: OSA = Obstructive sleep apnea; CSA = Central sleep apnea; AHI = Hypopnea apnea index; CI = Confidence interval; PPV = Positive predictive value; NPV = Negative predictive value; ROC = Receiver operating characteristic; *Severity of SDB (OSA and CSA according to AHI): >5 and <15 mild, >15 and <30 moderate, >30/hour severe.

**Figure 1:**
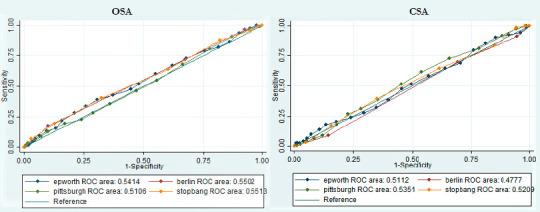
Operating characteristics curves (AUC-ROC) for the diagnosis of OSA and CSA in patients with cardiovascular disease. Notes: OSA = Obstructive sleep apnea; CSA = Central sleep apnea; ROC: Receiver operating characteristic.

Figure 1 shows a comparison of the receiver operating characteristic (ROC) curves with the respective area under the curve (AUC) for the diagnosis of OSA and CSA in patients with CVD; none of the scales showed a discrimination ability significantly better than the others. (OSA chi2=0.5595, CSA chi2=0.2585).

Table 4 shows the different cut-off values evaluated for each test in the population with CVD. For the ESS the best cut-off point value was found to be greater than 9, with usual value>11, both for screening OSA (S: 60%, E: 45%) and CSA (S: 52, E: 51%) For the Berlin questionnaire, the cut-off value was similar to the usual >2; for OSA (S: 72%, E: 33%) and CSA (S: 70%, E: 31%). For the STOP-bang questionnaire, the best cut-off value was a score =3; OSA (S: 88, E: 18) and CSA (S: 64.8, E: 38.6). For the PSQI, a similar cut-off value was found >5, for OSA (S: 80%, E: 22%) and CSA (S: 83.6%, E: 15.2%).

## DISCUSSION

The operating characteristics for the SDB the highest S was for the PSQI; for moderate to severe OSA it was (79.2%), followed by Berlin (54.2%), ESS (49.5%), and STOP-bang (26.4%). For diagnosis of moderate to severe OSA, STOP-bang showed E 90.8%, followed by ESS (47.1%), Berlin (31.1%), and PSQI (20%). Our results show that, when evaluating the operating characteristics of the questionnaires similar to that reported in the literature, no questionnaire shows good diagnostic performance when used by itself; all had AUC of 0.6 or less. This is consistent with previous results where, for example, for ESS the results have been poor in their ability to screen OSA (AUC 0.56, accuracy of 51-59% for the cutoff AHI=5) and have often shown better specificities than sensitivities. Although many of the studies in this regard have shown to be of poor quality or in highly selected populations, the above implies that, although the availability of the gold standard is limited in many regions, for now it is not possible to recommend the questionnaires or algorithms of prediction as the only diagnostic method^20, 22^.

The frequency of OSA in the population with and without CVD in this study was about 43% and for CSA of 19 and 10%, respectively. Our results are in agreement with what is described in the literature; in patients with heart failure for example, the reported prevalence of OSA varies widely^23^ according with the cut-off point of AHI employed and the association with CVD (For AHI>5 a prevalence of 55 an 87% is described in the low and high risk groups, respectively)^20^: in general, it is higher than those reported for the general population. According to a cross-sectional analysis of sleep heart health study, the presence of OSA with an AHI=11 confers a relative increase of 2.38 times in the probability of having heart failure independent of other factors^24^.

In our results, the high frequency of CSA found in a population with and without CVD is striking, in contrast to what is reported in literature. These changes can be explained both by altitude (66% of the population was from Bogota) and by the presence of heart failure; in the CVD group where the majority were men and with more comorbidities. Several studies done at high altitudes indicate that an increase in central apneas occurs even among healthy individuals and in the same way, AHI is higher for individuals with OSA, and obstructive events convert to predominately central events at altitude^25^; likewise, a study carried out in the city of Bogotá in patients with decompensated heart failure showed that all the patients had OSA, most were severe, with the presence of central sleep apneas^26^. On the other hand, the frequency of SDB was higher in the group of men, which is similar to previous reports.

In this study, for the detection of SDB, in general, the highest S (about 81%) was found for PSQI. It was validated in Colombia and an S of 89% and E of 86% have been described^27^. These results are expected given the usefulness of this test to assess overall sleep quality. The Berlin questionnaire showed moderate S for the diagnosis of OSA and CSA (61% and 63.5%) with poor E and better performance in the group of patients with CVD (S: 72%-70%). Polanía-Dussan et al. (2013)^17^ found a S of 87%, E of 70%, AUC of 0.78 in their study validating Berlin test for Colombia, which contrasts with our results (AUC around 0.5). The above is partly explained by the population included in their study, most patients attending sleep laboratories were from the city of Bogotá. Other studies have not shown good performance especially for AHI=5 as the cut-off value (S of 76% and E of 45%) with similar findings to ours, although with high methodological variability^20^. It is important to note that some studies have shown that, for example, classic OSA symptoms, such as drowsiness, snoring, and apnea, are reported more frequently in men, while fatigue, initial insomnia, depression, and headaches are more common in the women^12, 13, 28^; in the same way, comorbidities can produce changes in symptoms that are similar to the symptoms of OSA and then affect the performance of diagnostic test, which in turn can produce false positive results^28^.

In contrast to previously reported studies, the STOP-bang questionnaire showed a better E and low sensitivity for the diagnosis of OSA and CSA (82.1% and 84.9) in the group of patients with CVD, followed by ESS (55%). On the contrary, it showed poor performance in the group of patients without CVD. Previously, good performance was described in mainly perioperative patients with an S of 84%, 93% and 100% for AHI > of 5, 15 and 30, respectively, and generally lower E (47% for moderate OSA and 37% for severe OSA)^19^; the different results to those reported in the literature are probably due to the fact that the STOP-bang was developed in the surgical population and our study included patients with CVD and also the general population without CVD; many of them from high altitude.

**Table 4:** Areas under the curve (AUC) for each test in the diagnosis of OSA and CSA.

	OSA			CSA		
Test	AUC	CI 95%	Cut-off value	AUC	CI 95%	Cut-off value
Epworth	0.54	0.496 0.586	>9	0.51	0.454 0.568	>9
Berlin	0.55	0.509 0.590	>2	0.47	0.427 0.527	>2
Pittsburgh	0.51	0.465 0.555	>5	0.53	0.480 0.590	>5
STOP-bang	0.55	0.507 0.595	>3	0.52	0.465 0.576	>3

**Notes:** OSA = Obstructive hypopnea sleep apnea syndrome; CSA = Central sleep apnea; AUC: Areas under the curve; CI = Confidence interval.

In 2017, a meta-analysis was published describing the diagnostic performance for the Berlin, STOP-bang, stop and ESS questionnaires for detection of OSA according to the AHI; they concluded that STOP-bang is a more accurate tool with better diagnostic S and OR to detect OSA and could be used for early diagnosis in clinical settings. However, it should be borne in mind that age, gender difference, BMI and the presence of comorbid conditions in the participants are factors that affect the accuracy of screening tools and should be considered when applying theses questionnaires, as it is recognized that there is a high probability of bias due to heterogeneity in the studied populations^29^. A systematic review carried out in Canada (2010)^30^ also describes that there is inconsistency in the accuracy of the tests due to the heterogeneity of the designs (population, type of questionnaire, and validity) and highlight the usefulness of stop and STOP-bang for screening of OSA in surgical population due to its better methodological quality and ease of use.

The performance of screening questionnaires for identifying OSA in populations with increased cardiovascular risk is not yet fully established; knowing the importance of SDB in cardiovascular outcomes in our study, we specifically evaluated the performance of tests in patients with CVD and we assessed for the existence of a possible better cut-off score for each questionnaire; only for the ESS had a slightly lower cut-off value as compared to the previously established value (greater than 9) for the diagnosis of OSA and CSA. A study published in 2013, evaluating the performance of ESS for the diagnosis of OSA (AHI>5), strikingly found high E (82.7%, 95% CI: 77.3-87.3) for ESS and moderate S (61.6%, 95% CI: 59.3-63.9) with cut-off values >9 for men and 6 for women^31^. Our results imply that in patients with CVD being at high risk, whenever using this scale as part of the initial assessment, a lower cut-off value increasing S should be considered. However, there is no doubt that these patients should be evaluated through the golden standard.

Specifically, within the group of patients with coronary heart disease in our study, a high frequency of SDB (76%) was found, mostly in men, with AHI between 5 and 15 and >30. For the diagnosis of OSA and CSA, the best S in this group was shown by the PSQI and the best E was the STOP-bang questionnaire, for CSA with slightly higher values. Some authors have explored the use of questionnaires such as Berlin^32, 33^ and ESS^34, 35^ as a screening tool for OSA when this diagnostic suspicion is found alongside coronary risk. However, these studies have not taken the gold standard into account.

As limitations, it is a retrospective analysis study with the possibility of bias; additionally, the data did not allow discriminating by subgroups because the sample size was not calculated for this purpose. However, our study has strengths: the number of patients included is considerable; in addition, our results provide knowledge regarding the performance of screening tests in a sample of population from three cities being representative of different altitudes, including the general population and those with CVD.

Although the prevalence of SDB and specifically OSA is high and its association with CVD is clear, the questionnaires used for screening have poor operating characteristics. Despite the insufficient availability of sleep laboratories in many regions, according to the results of this study and previous evidence, it is not possible to recommend questionnaires or clinical prediction rules as a single or independent diagnostic test to replace the polysomnogram, since they neither rule out or confirm the diagnosis. However, the use of screening tests that, although imperfect, could improve decision-making processes regarding an initial diagnostic strategy could be justified. Likewise, taking into account the influence of altitude on sleep physiology, it is necessary to understand the performance of different diagnostic tests in populations with different altitudes. High-risk patients with CVD, however, must prioritarily be assessed by the gold standard. In general, more studies are required to evaluate the performance of new proposals (combination of tests, series, parallel studies) or prediction models in populations that are not highly selected, also seeking to reduce the likelihood of inherent biases.

## Appendix

**Supplementary Table 1:** Operating characteristics of screening tests in patients with coronary heart disease.

OSA				
Prevalence 44% (40-49.3) CI 95%	Epworth	Berlin	STOP-bang	Pittsburgh
Sensitivity%	48.4 (41.1-55.8)	73.4 (66.5-79.6)	11.2 (7.05-16.6)	80.3 (73.9-85.7)
Specificity %	53.6(47-60.1)	32.8(26.8-39.2)	83.4 (78-87.9)	21.3 (16.2-27.1)
PPV %	45.5 (38.5-52.7)	46.6 (40.8-52.5)	35 (23.1-48.4)	44.9 (39.5-50.4)
NPV%	56.5 (49.7-63.1)	60.6 (51.6-69.2)	54 (48.7-59.2)	57.5 (46.4-68)
ROC area	0.51 (0.46-0.55)	0.53 (0.48-0.57)	0.47 (0.44-0.50)	0.50 (0.46-0.54)
**Prevalence 19% ( 16-23.2) CSA**				
Sensitivity%	49.4 (38.1-60.7)	70.4 (59.2-80)	14.8(7.9-24.4)	80.2 (69.9-88.3)
Specificity %	53.2 (47.8-58.6)	30.1(25.3-35.3)	86(81.8-89.5)	20.8 (16.6-25.5)
PPV%	20 (14.7-26.2)	19.3 (14.9-24.2)	20 (10.8-32.3)	19.3 (15.3-24)
NPV%	81.6 (75.9-86.5)	81.1 (73.2-87.5)	81(76.6-84.9)	81.6 (71.9-89.1)
ROC area	0.51 (0.45-0.57)	0.50 (0.44-0.55)	0.50 (0.46-0.54)	0.50 (0.45-0.55)

**Notes:** OSA = Obstructive hypopnea sleep apnea syndrome; CSA = Central sleep apnea; CI = Confidence interval; PPV = Positive predictive value; NPV = Negative predictive value; ROC = Receiver operating characteristic.
